# Lessons from the Impact of War in Ukraine on Combatants’ Mental Health during the Last Decade

**DOI:** 10.1192/j.eurpsy.2023.407

**Published:** 2023-07-19

**Authors:** A. Haydabrus, L. Giménez-Llort

**Affiliations:** ^1^Department of Neurology, Psychiatry, Narcology and Medical Psychology, School of Medicine, V.N. Karazin Kharkiv National University, Kharkiv, Ukraine; ^2^Department of Psychiatry and Forensic Medicine, School of Medicine, Universitat Autònoma de Barcelona, Barcelona, Spain

## Abstract

**Introduction:**

The threat and preservation of the territorial integrity of Ukraine are not new issues. During the last decade, peacetime (Peace, until 2013) was disrupted by active hostility (AH, 2014–2015) and trench warfare (TW, 2016–2021). War exert acute and chronic impacts on mental health, may be a substrate for mental health disorders, especially worrisome since, today, the large-scale conflict has demanded the recruitment of adult civilians to defend and fight alongside armed forces troops.

**Objectives:**

The analysis aimed to unveil the impact of those conflicts on the mental health of the army and help us to anticipate risk factors (ranks, time period) and need for resources (admissions and days of hospitalization per time period, rank and disease).

**Methods:**

A retrospective cross-sectional analysis of an anonymized part of the internal database included 3995 anonymized records.Data are expressed as the frequency (%), fold-increase, or mean ± SEM. Chi-square analysis and ANOVA with Bonferroni post hoc correction were performed with Jamovi.

**Results:**

The temporal distribution of admissions (Figure 1) showed a 6.97 (AH) and 3.62 (TW) [5.02 (TW1, 2016–2017), 3.91 (TW2, 2018–2019), and 1.95 (TW3, 2020–2021)] fold increase per year compared to peacetime. The most frequent mental health problems, accounting for 76.1% of cases, were ‘anxiety, dissociative, stress-related, somatoform and other nonpsychotic disorders’ (F40-F48, ANXd, 40.1%) and ‘mental and behavioral disorders due to psychoactive substance use’ (F10–F19, PSUd, 36.0%). ‘Reaction to severe stress and adjustment disorders (F43, 76.5%) and ‘Alcohol-related disorders (F10, 89.3%) were the predominant mental health disorders, respectively.

The ICD-10 category depended on the war period (Figure 2), with peacetime to TW2 accounting for 90% of cases. ‘ANXd’ were the main mental health problem in any period, with 61.8% of cases occurring in peacetime. PSUd, residual in peacetime 6.1%, reached their peak in active hostility (47.4%), with 97.9% of ‘Alcohol-related disorders’ as the cause of these admissions, which could agree with the use of alcohol serving as a coping mechanism in front traumatic events. In trench warfare, PSUd decreased (TW1, 39.2%; TW2, 25.1%).

Hospital stays for people with ANXd or PSUd lasted at least one month in peacetime but significantly decreased in war periods (Figure 3). This could be explained by a ‘need for free beds effect’ and the distribution of admissions by ranks.

**Image:**

**Figure:**
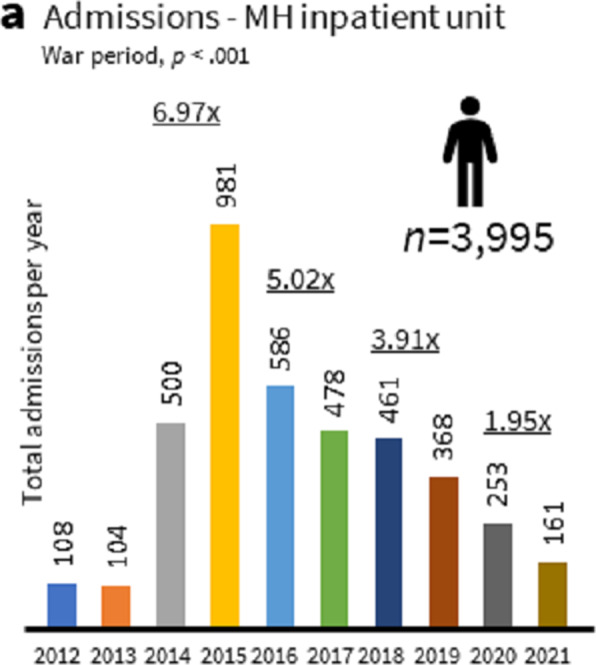

**Image 2:**

**Figure:**
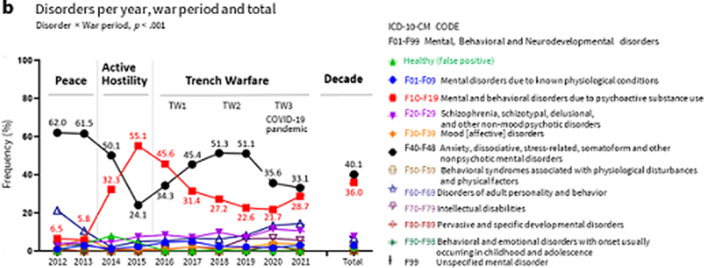

**Image 3:**

**Figure:**
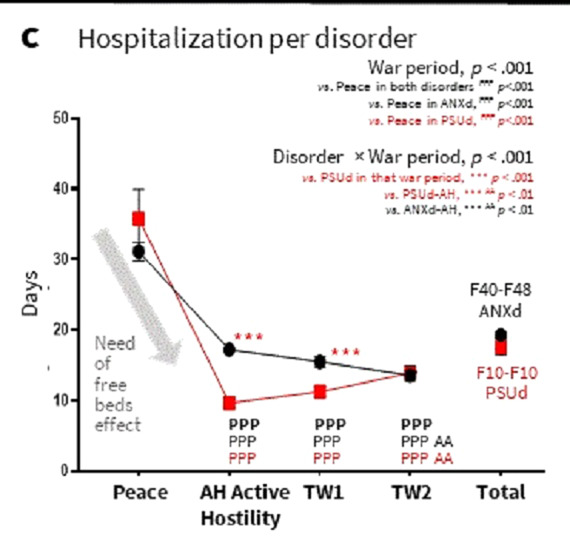

**Conclusions:**

-

The dominance of ANXd, mainly among professional soldiers and high ranks, points to the need for rank-tailored psychological training in skills to reduce the ANXd burden.

-

The large number of PSUd in nonprofessional soldiers during wartime dictates the need to strengthen the selection of military personnel.

-

Hospitalizations in military operations are heterogeneous and depend on the military rank.

**Disclosure of Interest:**

None Declared

